# The 4th Bologna Winter School: Hot Topics in Structural Genomics

**DOI:** 10.1002/cfg.314

**Published:** 2003-07

**Authors:** Rita Casadio

**Affiliations:** Department of Biology/CIRB University of Bologna Via Irnerio 42 Bologna 40126 Italy

## Abstract

The 4th Bologna Winter School on Biotechnologies was held on 9–15 February 2003 at the University of Bologna, Italy, with the specific aim of discussing recent developments in bioinformatics. The school provided an opportunity for students and scientists to debate current problems in computational biology and possible solutions. The course, co-supported (as last year) by the European Science Foundation program on Functional Genomics, focused mainly on hot topics in structural genomics, including recent CASP and CAPRI results, recent and promising genomewide predictions, protein–protein and protein–DNA interaction predictions and genome functional annotation. The topics were organized into four main sections (http://www.biocomp.unibo.it).


**Comparative and Functional Genomics**
*Comp Funct Genom* 2003; **4**: 394–396.
Published online in Wiley InterScience (www.interscience.wiley.com). **DOI:** 10.1002/cfg.314

**Conference Report**

### The 4th Bologna Winter School: hot topics in structural genomics†

Rita Casadio*
*Department of Biology/CIRB, University of Bologna, Via Irnerio 42, 40126 Bologna, Italy*

**Correspondence to:*
*Rita Casadio, Department of Biology/CIRB, University of Bologna, Via Irnerio 42, 40126 Bologna, Italy.*
*E-mail: casadio@alma.unibo.it*

Received: 3 June 2003
Revised: 5 June 2003
Accepted: 5 June 2003

### Abstract

**The 4th Bologna Winter School on Biotechnologies was held on 9–15 February 2003 at the University of Bologna, Italy, with the specific aim of discussing recent developments in bioinformatics. The school provided an opportunity for students and scientists to debate current problems in computational biology and possible solutions. The course, co-supported (as last year) by the European Science Foundation program on Functional Genomics, focused mainly on hot topics in structural genomics, including recent CASP and CAPRI results, recent and promising genome-wide predictions, protein–protein and protein–DNA interaction predictions and genome functional annotation. The topics were organized into four main sections (http://www.biocomp.unibo.it). Published in 2003 by John Wiley & Sons, Ltd.**

### Predictive methods in structural genomics

- Contemporary challenges in structure prediction and the CASP5 experiment (John Moult, University of Maryland, Rockville, MD, USA).
- Contemporary challenges in structure prediction (Anna Tramontano, University 'La Sapienza', Rome, Italy).
- Prediction of protein structure and function at the genomic scale (Jeffrey Skolnick, Buffalo Center of Excellence in Bioinformatics, Buffalo, NY, USA).
- Advanced automated machine learning approaches (David Jones, University College, London, UK).
- Fully automated *ab initio* protein structure prediction (Chris Bystroff, Rensselaer Polytechnic Institute, Troy, NY, USA).
- Automatic fold recognition prediction (Daniel Fischer, Ben Gurion University, Be'er Sheva, Israel).

† This article is an adaptation of the Conference Report on this workshop (published to satisfy the requirements of the ESF funding) previously published on the Integrated Approaches for Functional Genomics website.

### Predictive methods in functional genomics

- Prediction of protein function (Arthur Lesk, University of Cambridge, Cambridge, UK).
- Microarray data analysis and mining (Raffaele Calogero, University of Torino, Torino, Italy).
- Prediction of protein function and protein networks (Søren Brunak Technical University of Denmark, Lyngby, Denmark, and Alfonso Valencia, National Centre of Biotechnology, Cantoblanco, Spain).

### Prediction of membrane protein structure

- The prediction of membrane protein topology (Gunnar von Heijne, University of Stockholm, Stockholm, Sweden, and Stephen White, University of California, Irvine, CA, USA).
- Application of structural genomics tools to fishing for new membrane proteins (Rita Casadio, University of Bologna, Italy).

Published in 2003 by John Wiley & Sons, Ltd.

### Prediction of protein–protein and protein–DNA interaction

- The CAPRI experiment and the prediction of protein–protein interactions (Joel Janin, LEBS, CNRS, Gif sur Yvette, France).
- Prediction of protein complexes based on evolutionary information (Patrick Aloy, EMBL Heidelberg, Germany).
- Prediction of protein–DNA complexes (Sue Jones, European Bioinformatics Institute, Hinxton, UK).
- Prediction of functional patches in proteins (Manuela Helmer Citterich University of Tor Vergata, Rome, Italy, and Nir Ben Tal, Tel Aviv University, Tel Aviv, Israel).

### What can we predict in the post genomic era? Is it feasible to perform large-scale prediction on entire genomes? How can we cope with predictive tools?

The CASP (critical assessment of protein structure prediction techniques) experiment reports on how different methods perform in specific predictive tasks. The results had been debated just 2 months earlier at the CASP5 meeting in Asilomar on 1–5 December 2002 (**http://predictioncenter.llnl.gov/casp5/Casp5.html**). Apparently, as explained by its organizer (John Moult) and one of its assessors (Anna Tramontano, assessor of the homology building section), one cannot state that a particular method is the best. There are several excellent tools that, when integrated into meta-servers (as described by Daniel Fisher), can perform fold recognition in a very satisfactory way. Therefore, integrated knowledge will help us in solving the folding problem also on a genome-wide scale. Jeffrey Skolnick is modelling most of the protein content of presently available known proteomes, including some functional properties, based on a sequence-to-structure-to-function paradigm. With a very low rate of false positives, his tools can also predict folds of some 30–50% of the proteins. Machine learning approaches form the basis of the most successful tools for prediction of structural features, including secondary structure of proteins, as David Jones reported. Chris Bystroff explained that proteins can also be predicted *ab initio*, provided that an HMM-based method, predicting protein contact maps, is used.

### What about predicting function?

Raffaele Calogero discussed DNA microarray technology, a high-throughput method for gaining information on gene function. However, genomes can only be completely annotated when we can predict the function, possibly starting from the sequence. This is presently a really difficult and challenging task, as Arthur Lesk pointed out in his talk. Søren Brunak has used a suite of programs, integrating predictions of different structural and functional properties, to successfully address this problem. Alfonso Valencia described how predictions are of a quality similar to the experimental data, when different interacting protein networks (at the basis of systems biology, and obtained using both theoretical and experimental approaches) are compared. Also, the interactions detected by more than one method have a substantially higher confidence.

### Good results are being achieved in the case of globular proteins, but when it comes to membrane proteins, the situation is much worse

Less than 1% of PDB structures are membrane proteins and, as a result, building by homology is hampered by the paucity of examples, except possibly in the case of outer membrane proteins. The $\beta$-barrel architecture is rather conserved and the changes that are seen are in the number of antiparallel beta strands. Once this is predicted, a 3D model can be computed with a threading procedure (as explained in my own presentation). In the case of inner membrane proteins, one is mainly left with the prediction of topological models. Gunnar von Heijne presented some recent advances in the identification of membrane protein topology and its prediction with bioinformatic methods. Stephen White described two Web-based tools, MPEx (**http://blanco.biomol.uci.edu/mpex**) and MPtopo (**http://blanco.biomol.uci.edu/mptopo**), which are based on chemical-physical properties of transmembrane $\alpha$-helices and 'designed to help experimentalists explore the topology of membrane proteins of unknown 3D structure'.

Published in 2003 by John Wiley & Sons, Ltd.

### Can we predict protein–protein and protein–DNA interactions?

Joel Janin described the CAPRI (critical assessment of predicted interactions; **http://capri.ebi.ac.uk**) experiment. Inspired by CASP, it similarly accepts predictions of proteins interacting in complexes, whose structures are known only after the submission is closed. The results of the last edition (which were evaluated by Shoshana Wodak and Raul Mendez, Free University of Brussels, Belgium) show significant success on some of the targets. However, the predictions failed on other targets, and Joel believes that progress is necessary 'in the score functions, and in the way docking procedures handle conformation changes and non-structural information, before large-scale predictions of protein–protein interactions can be made reliably' (**http://www.biocomp.unibo.it/school/html2003**). Sue Jones detailed some interesting features of protein–DNA complexes and Patrick Aloy explained how protein interactions can be predicted through tertiary structure. If we know the structure, we can also predict functional patches on the protein surface, as described in the talks of Nir Ben Tal and Manuela Helmer-Citterich.

Finally, a round table discussion (chaired by Anna Tramontano and Arthur Lesk) focused on the relevance of bioinformatics in different experimental fields, including membrane protein topology characterization (Alessandro Desideri, University of Tor Vergata, Rome, Italy, and Richard Wagner, University of Osnabrück, Germany), protein structure determination with NMR (Henriette Molinari, University of Verona, Italy), and protein folding studies with atomic force microscopy (Bruno Samori, University of Bologna, Italy).

The take home message was that bioinformatics can offer solutions to problems, provided that theoreticians and experimentalists work in close collaboration to benefit from both a computational and an experimental approach.

### Acknowledgements

The school was made possible thanks to the invaluable help of Piero Fariselli, Pier Luigi Martelli, Ivan Rossi, Gianluca Tasco, Emidio Capriotti, Luca Malaguti, Remo Calabrese and Mario Compiani, all at the Biocomputing Unit, and the precious collaboration of Professor Lanfranco Masotti (Co-chairman of the School) at the Department of Biochemistry, University of Bologna. Other supporting organizations were: the University of Bologna, the Interdepartmental Centre for Biotechnological Research (CIRB), the Consorzio Interuniversitario Biotechnologie (CIB), the Istituto Nazionale Biostrutture Biosistemi (INBB), the Marino Golinelli Foundation, the FIRB project, the Italian National Academy of Lincei, the Italian National Council for Research (CNR), the Italian Association for Research in Biophysics and Computational Biology (AIRBBC), and all of the students.

